# Scale-Consistent and Temporally Ensembled Unsupervised Domain Adaptation for Object Detection

**DOI:** 10.3390/s25010230

**Published:** 2025-01-03

**Authors:** Lunfeng Guo, Yizhe Zhang, Jiayin Liu, Huajie Liu, Yunwang Li

**Affiliations:** 1 School of Mechanical and Electrical Engineering, China University of Mining and Technology (Beijing), Beijing 100083, China; bqt2000401001@student.cumtb.edu.cn (L.G.); bqt2000401002@student.cumtb.edu.cn (Y.Z.); yunwangli@cumtb.edu.cn (Y.L.); 2 Mining University (Beijing) Inner Mongolia Research Institute, Ordos 017000, China; 3 Key Laboratory of Smart Mining and Robotics, Ministry of Emergency Management, Beijing 100083, China; 4 China Academy of Safety Science and Technology, Beijing 100083, China; 5 Suzhou Automotive Research Institute (Wujiang), Tsinghua University, Suzhou 215200, China; liuhuajie@tsari.tsinghua.edu.cn

**Keywords:** object detection, autonomous driving, unsupervised domain adaption

## Abstract

Unsupervised Domain Adaptation for Object Detection (UDA-OD) aims to adapt a model trained on a labeled source domain to an unlabeled target domain, addressing challenges posed by domain shifts. However, existing methods often face significant challenges, particularly in detecting small objects and over-relying on classification confidence for pseudo-label selection, which often leads to inaccurate bounding box localization. To address these issues, we propose a novel UDA-OD framework that leverages scale consistency (SC) and Temporal Ensemble Pseudo-Label Selection (TEPLS) to enhance cross-domain robustness and detection performance. Specifically, we introduce Cross-Scale Prediction Consistency (CSPC) to enforce consistent detection across multiple resolutions, improving detection robustness for objects of varying scales. Additionally, we integrate Intra-Class Feature Consistency (ICFC), which employs contrastive learning to align feature representations within each class, further enhancing adaptation. To ensure high-quality pseudo-labels, TEPLS combines temporal localization stability with classification confidence, mitigating the impact of noisy predictions and improving both classification and localization accuracy. Extensive experiments on challenging benchmarks, including Cityscapes to Foggy Cityscapes, Sim10k to Cityscapes, and Virtual Mine to Actual Mine, demonstrate that our method achieves state-of-the-art performance, with notable improvements in small object detection and overall cross-domain robustness. These results highlight the effectiveness of our framework in addressing key limitations of existing UDA-OD approaches.

## 1. Introduction

Recent advancements in deep learning have led to significant progress in object detection across various applications, including autonomous driving, security surveillance, and medical imaging [1]. However, object detection models trained on a source domain often perform poorly when directly applied to a target domain due to domain shift [2]. These shifts arise from differences in object appearance, background, or scale, severely limiting the generalizability of detection models. To address this challenge, unsupervised domain adaptation for object detection (UDA-OD) techniques leverage unlabeled target domain data to reduce the domain shift and improve detection performance [3].

Recent advances in UDA-OD methods based on the mean teacher framework have garnered considerable attention [4,5,6]. These methods employ a teacher model to generate pseudo-labels, which are then used to guide a student model’s learning process, progressively improving detection performance in the target domain. However, these methods still have limitations in pseudo-label selection and handling object scale variations.

A major limitation in existing methods is their inadequate handling of object scale variations. While large-scale objects are easier to detect, small-scale objects are often ignored, leading to degraded detection performance. Although some methods attempt to mitigate scale differences through cross-domain multi-scale feature alignment [7], they often fail to ensure cross-scale consistency in target domain. Other approaches use full-scale gated fusion modules [8] to enhance multi-scale adaptation but introduce high computational complexity during inference, limiting practical deployment.

Additionally, current methods mainly depend on classification confidence to select pseudo-labels, without considering the accuracy of bounding box localization. High classification confidence does not always equate to precise localization [9], resulting in training with bounding boxes that have large errors. To address this issue, some methods introduce multi-input perturbations [10] or modify the output head to additionally predict variance [11] as a measure of bounding box quality. However, these approaches incur high computational overhead, reducing inference efficiency.

To this end, we propose a UDA-OD method utilizing scale consistency and temporal ensemble mechanisms. Specifically, we adopt the mean teacher framework and supervise the student model with multi-scale inputs to ensure consistent detection results across different resolutions, enhancing small-scale object detection. Furthermore, we employ contrastive learning to ensure feature consistency for objects of the same class across different scales and different data augmentation, thereby improving intra-class feature alignment and inter-class separation. Finally, we introduce a pseudo-label selection strategy based on temporal ensemble, combining temporal localization consistency with classification confidence to ensure high-quality pseudo-labels while avoiding the high computational costs of multi-perturbation predictions.

The most relevant mean-teacher-based UDA-OD methods to our work are Adaptive Teacher (AT) [4] and its extension Contrastive Mean Teacher (CMT) [5]. AT introduces a teacher model to guide the student model, significantly improving UDA-OD performance. Our work builds on this baseline, adding a scale consistency and a temporal ensemble pseudo-label selection strategy. While CMT also computes contrastive loss for enhanced UDA-OD performance, its student model only accepts inputs from a single scale, failing to compute feature contrast loss across scales and limiting its performance with multi-scale instances.

Our main contributions are summarized as follows:

- We propose a cross-scale prediction consistency strategy to enhance model adaptability to objects of varying scales, focusing on improving small object detection. Additionally, contrastive learning is employed to strengthen intra-class feature consistency.
- We introduce a pseudo-label selection strategy that integrates temporal localization consistency with classification confidence to improve the quality and stability of pseudo-labels.
- Extensive experiments on diverse object detection datasets show that our method outperforms state-of-the-art UDA-OD approaches, particularly in addressing object scale variations and optimizing pseudo-label selection.

The remainder of this paper is organized as follows: Section 2 reviews related work on domain adaptation and object detection methods. Section 3 presents the proposed approach, including the scale consistency and temporal ensemble pseudo-label selection strategies. Section 4 details the experimental setup and analyzes the results, demonstrating the efficacy of our method. Finally, Section 6 provides concluding remarks.

## 2. Related Work

### 2.1. Object Detection

Object detection has significant advancements, with advances in deep learning techniques. Early methods such as Faster R-CNN [12], SSD [13], and YOLO [14] set the foundation for accuracy–speed balance. Faster R-CNN uses a two-stage detection method coupled with a Region Proposal Network (RPN), while SSD and YOLO employ a single-stage design to enhance inference speed. Subsequently, anchor-free (AF) models like CornerNet [15] and FCOS [16] were proposed, removing predefined anchor boxes to simplify detection and reduce computation.

Recently, end-to-end object detection has become a hot research topic, directly predicting detection boxes. DETR [17] is a typical representative, capturing object relationships through Transformer’s self-attention. Improved versions of Deformable DETR [18] and DINO [19] further enhance speed and performance through deformable attention. Another approach is Sparse R-CNN [20], achieving end-to-end detection via learnable candidate frames.

In autonomous driving, object detection is crucial for subsequent planning and control. Systems must detect multiple targets, including vehicles, obstacles, lane lines, traffic signs, etc. [21]. However, existing object detection methods usually rely on large-scale annotated data for supervised learning and assume similar feature distributions for training and test data. This assumption often does not hold in practice, especially for autonomous driving, where differences in weather conditions, sensors, and city can significantly affect model generalization.

Domain adaptation techniques are thus crucial for cross-domain applications with limited target domain annotation data, helping maintain performance across environments and scenarios and improving adaptability and reliability in complex tasks like autonomous driving.

### 2.2. Unsupervised Domain Adaptation for Object Detection

UDA-OD aims to improve the model’s object detection performance in the target domain by adapting models from a labeled source domain to an unlabeled target domain with different data distributions [3]. This is especially useful when labeled data are difficult to obtain in the target domain. A common strategy in UDA-OD is adversarial feature alignment, where a domain discriminator encourages the model to make source and target features indistinguishable [2]. This approach has been extended to multi-level alignment, with global and local feature alignment techniques that enhance adaptation performance [7].

For more challenging cases of domain shift, image translation methods [22,23] are sometimes used to make target images resemble the source domain, thereby reducing visual discrepancies and improving model transferability across environments.

Another approach is pseudo-labeling, where a source-trained model generates labels for the target domain data [24]. Mean teacher frameworks [4,5], which use a consistency-based approach between a teacher and a student model, further improve this by gradually refining pseudo-labels over time. By leveraging both labeled source data and unlabeled target data, mean teacher frameworks stabilize training and have become a foundation in recent UDA methods for object detection.

## 3. Method

**Figure 1:**
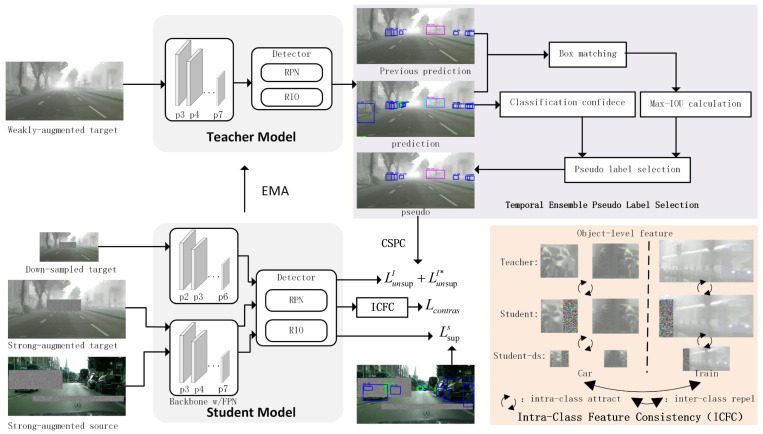
Overview of the proposed method for the UDA-OD task. The figure illustrates the teacher–student architecture, where the teacher model generates pseudo-labels based on weakly augmented target domain images, and the student model is trained using both downsampled and strongly augmented inputs. CSPC is enforced by supervising the student model with inputs of different resolutions, improving detection of objects at various scales. Temporal ensemble is employed for robust pseudo-label selection, combining classification confidence and box matching based on Intersection over Union (IoU) to ensure high-quality pseudo-labels. Additionally, the ICFC module aligns object-level features across scales and augmentations, utilizing contrastive learning to ensure intra-class attraction and inter-class repulsion, enhancing the consistency of object representations.

As depicted in Figure 1, we propose a UDA-OD method based on the mean teacher framework, integrating scale consistency (SC) and Temporal Ensemble Pseudo-Label Selection (TEPLS) to enhance object detection performance in cross-domain settings. Specifically, the teacher–student architecture is employed, where the teacher model generates pseudo-labels from weakly augmented target domain images, while the student model is trained with both downsampled and strongly augmented versions of the target images. To handle object scale variations, we introduce Cross-Scale Prediction Consistency (CSPC), where the student model is supervised using inputs of different resolutions to improve detection across various object scales. Additionally, we employ Intra-Class Feature Consistency (ICFC) through contrastive learning to align object features across scales and augmentations, enhancing intra-class alignment and inter-class separation. To ensure robust domain adaptation, we also introduce a temporal ensemble mechanism for pseudo-label selection, combining classification confidence with bounding box consistency over time to improve pseudo-label quality without incurring high computational costs.

### 3.1. Problem Formulation

In the context of UDA-OD, we aim to adapt an object detection model from a labeled source domain $D_{S}={\left\{\left(x_{i}^{s},y_{i}^{s}\right)\right\}}_{i=1}^{n_{s}}$ to an unlabeled target domain $D_{T}={\left\{x_{j}^{t}\right\}}_{j=1}^{n_{t}}$. Here, $x_{i}^{s}$ and $x_{j}^{t}$ represent images from the source and target domains, respectively, while $y_{i}^{s}$ denotes the labeled annotations of the source domain images, including bounding box coordinates $b_{i}^{s*}$ and class labels $c_{i}^{s*}$. The target domain only contains unlabeled images, and our objective is to enhance the performance of the object detection model in the target domain without utilizing labeled annotations.

### 3.2. Scale Consistency

Existing UDA-OD methods struggle to handle variations in object scales, frequently overlooking small-scale objects that are crucial for accurate detection. These methods often generate incomplete pseudo-labels, particularly for small objects, which can degrade overall detection performance. To address this limitation, we introduce a comprehensive method that combines cross-scale prediction consistency and intra-class feature consistency. This approach helps the model learn consistent feature representations across different scales and data augmentations, thereby improving its generalization capabilities, especially for small objects.

#### 3.2.1. Cross-Scale Prediction Consistency

We use Faster R-CNN [12] as the detection framework, with ResNet-50 FPN as the backbone network. The student model takes in two inputs: an original resolution image of the target domain, denoted as $x^{t}$, and a downsampled version $x^{t\prime }$, created by halving the original pixel dimensions (e.g., 1024 × 1024 to 512 × 512 pixels). This downsampling ratio aligns with the spatial resolution differences between adjacent FPN layers, enabling effective alignment of the feature maps of $x^{t}$, ${\left\{P_{l}^{I}\right\}}_{l=3}^{7}$, generated by the P3–P7 layers of the FPN, with the feature maps of $x^{t^{\prime }}$, ${\left\{P_{l}^{I\prime }\right\}}_{l=2}^{6}$, generated by the P2–P6 layers. To ensure that the feature layers from the downsampled image align with the index of the feature layers from the original image, $P_{2}^{I\prime }$ through $P_{6}^{I\prime }$ are mapped to $P_{3}^{I^{*}}$ through $P_{7}^{I^{*}}$.

This alignment enables the same pseudo-label boxes to supervise both the original and downsampled images, facilitating more effective training of the student model. It simplifies the alignment of cross-scale features and ensures that the feature maps of the original resolution and downsampled resolution represent the same image regions at corresponding spatial locations. This improves consistency and robustness in multi-scale feature learning.

The teacher model produces pseudo-labels $\hat{y}$, which are used to compute the classification losses for both the Region Proposal Network (RPN) and Region of Interest (ROI) head. The RPN classification losses for the original and downsampled resolutions are defined as follows:

(1) $$L_{\mathrm{RPN}}^{I}=\frac{1}{N}\sum_{n=1}^{N}\mathrm{CE}\left(\mathrm{RPN}\left(P_{l}^{I}\right),\hat{y}\right),\;L_{\mathrm{RPN}}^{I^{*}}=\frac{1}{N^{*}}\sum_{n=1}^{N^{*}}\mathrm{CE}\left(\mathrm{RPN}\left(P_{l}^{I^{*}}\right)\hat{y}\right)$$

where *N* and $N^{*}$ denote the number of region proposals for each resolution, and CE(·) represents the cross-entropy loss. Similarly, the ROI classification losses are as follows:

(2) $$L_{\mathrm{ROI}}^{I}=\frac{1}{M}\sum_{m=1}^{M}\mathrm{CE}\left(\mathrm{ROI}\left(P_{l}^{I},\mathrm{RPN}\left(P_{l}^{I}\right)\right),\hat{y}\right),\;L_{\mathrm{ROI}}^{I^{*}}=\frac{1}{M^{*}}\sum_{m=1}^{M^{*}}\mathrm{CE}\left(\mathrm{ROI}\left(P_{l}^{I^{*}},\mathrm{RPN}\left(P_{l}^{I}\right)\right),\hat{y}\right)$$

where *M* and $M^{*}$ denote the number of ROIs for each resolution. These combined unsupervised losses defined as

(3) $$L_{\mathrm{unsup}}^{I}=L_{\mathrm{RPN}}^{I}+L_{\mathrm{ROI}}^{I},\;L_{\mathrm{unsup}}^{I^{*}}=L_{\mathrm{RPN}}^{I^{*}}+L_{\mathrm{ROI}}^{I^{*}}$$

guide the student model to align its predictions with the pseudo-labels generated by the teacher model across both resolutions.

By training on the same pseudo-labels at different resolutions, large objects become smaller after downsampling, thus increasing the number of pseudo-labels for small objects. This mechanism effectively expands the training samples for small objects and improves the overall detection performance for small objects.

#### 3.2.2. Intra-Class Feature Consistency

In addition to cross-scale prediction consistency, we introduce an intra-class feature consistency mechanism to reduce the model’s reliance on specific augmentations or feature scales, thereby enhancing generalization and small object detection. Specifically, the teacher model extracts features from strongly augmented images, while the student model extracts features from weakly augmented images at varying scales. These features are then aligned in the feature space through intra-class contrastive learning, ensuring consistent representation of same-class target features across different conditions.

To achieve this, we extract features from corresponding layers (e.g., layers 3 to 7) of the feature pyramids for both the teacher and student models. For each target domain image in a batch, the teacher model processes weakly augmented images to produce feature maps, denoted as ${\left\{P_{l}^{T,bat}\right\}}_{l=3}^{7}$, where bat∈{1,…,BAT} represents the image index in the batch, and BAT is the batch size. The bounding boxes generated by the teacher model for all target domain images in the batch are used to compute the contrastive loss. Through ROI-Align, we extract features corresponding to these bounding boxes from each layer of the feature maps, referred to as target object features. For the *l*-th feature map of the bat-th image, we extract target object features from the teacher model’s feature map $P_{l}^{T,bat}$, the student model’s original resolution feature map $P_{l}^{I,bat}$, and the downsampled resolution feature map $P_{l}^{I^{*},bat}$. These target object features are then L2-normalized and concatenated at the batch level:

(4) $$R_{l}^{v}=\overset{BAT}{\underset{bat=1}{⋃}}\mathrm{Norm}\left(\mathrm{ROIAlign}\left(P_{l}^{v,bat},{\hat{B}}^{bat}\right)\right),\;v\in \left\{T,I,I^{*}\right\}$$

where ${\hat{B}}^{bat}$ denotes the bounding box coordinates of the pseudo-labels predicted by the teacher model for the bat-th image. $R_{l}^{T}$, $R_{l}^{I}$, and $R_{l}^{I^{*}}$ represent the sets of target object features extracted from the teacher model, the student’s original resolution branch, and the downsampled resolution branch at layer *l*, respectively, with each set containing *N* features.

To ensure consistency of object features across different scales, we calculate a contrastive loss across corresponding feature layers, defined as follows:

(5) $$L_{\mathrm{contra}}=\sum_{l=3}^{7}\frac{1}{3N}\sum_{i=1}^{N}\sum_{v\in \{T,I,I^{*}\}}\frac{1}{\left|Pos(i)\right|}\sum_{p\in Pos(i)}-\log\frac{\exp(\mathrm{sim}\left(R_{i,l}^{v},R_{p,l}\right)/\tau )}{\sum_{a\in A(l)}\exp\left(\mathrm{sim}\left(R_{i,l}^{v},R_{a,l}\right)/\tau \right)}$$

where sim(a,b) denotes the similarity between feature vectors *a* and *b*, typically computed using cosine similarity. The temperature parameter τ controls the sharpness of the similarity distribution. The set A(l) represents all target object features in the current batch, defined as follows:

(6) $$A\left(l\right)=\{R_{j,l}^{v}|j=1,\ldots ,N,v\in \left\{T,I,I^{*}\right\}\}$$

which includes features for each pseudo-label class across the teacher model *T*, the student model’s original resolution branch *I*, and the downsampled resolution branch $I^{*}$, totaling 3N features. The set Pos(i) denotes positive samples that belong to the same class as sample *i*, defined as follows:

(7) $$Pos\left(i\right)=\{p|c_{p}=c_{i},p\neq i,p\in A\left(l\right)\}$$

This contrastive loss, applied at each layer of the feature pyramid, aligns ROI features of the same class across scales and augmentations, enhancing model robustness and small object detection.

By integrating CSPC and ICFC, the model learns stable and consistent feature representations, improving generalization and significantly enhancing detection performance in the target domain.

### 3.3. Temporal Ensemble Pseudo-Label Selection

Building on the improvements in object scale consistency, it is also crucial to enhance the quality and stability of pseudo-labels. Existing UDA-OD methods [4,5] primarily rely on classification confidence to select pseudo-labels, which poses two major issues. First, high classification confidence does not necessarily guarantee accurate localization, especially in scenarios with significant domain shifts, where the model may overfit certain visual features of the target object, leading to inaccurate localization. Second, bounding box regression is a continuous prediction task, lacking a natural confidence metric comparable to classification, which makes assessing localization quality challenging.

Based on these observations, we propose using temporal consistency as an alternative metric to evaluate localization quality. This design is based on a core hypothesis: for accurately localized objects, the predicted bounding box positions should remain relatively stable across different training stages. Temporal consistency thus provides a quality evaluation mechanism without incurring additional computational costs, avoiding the need to explicitly model localization uncertainty.

Specifically, assume that at the *t*-th iteration, for a target domain image $x_{j}^{t}$, the teacher model’s predictions are represented as follows:

(8) $$P_{\mathrm{cur}}={\left(b_{i}^{\mathrm{cur}},c_{i}^{\mathrm{cur}},s_{i}^{\mathrm{cls}}\right)}_{i=1}^{n}$$

where $b_{i}^{\mathrm{cur}}$ is the predicted bounding box coordinate, $c_{i}^{\mathrm{cur}}$ is the predicted class, and $s_{i}^{\mathrm{cls}}$ is the classification confidence score.

Additionally, we save the predictions for the same image from the previous epoch:

(9) $$P_{\mathrm{prev}}={\left(b_{k}^{\mathrm{prev}},c_{k}^{\mathrm{prev}},s_{k}^{\mathrm{cls}}\right)}_{k=1}^{m}$$

For each bounding box in the current predictions, we first find the set of bounding boxes from the previous epoch that have the same predicted class:

(10) $$B_{\mathrm{prev}}^{\left(c_{i}\right)}=\left\{b_{k}^{\mathrm{prev}}|c_{k}^{\mathrm{prev}}=c_{i}^{\mathrm{cur}}\right\}$$

We then compute the Intersection over Union (IoU) between $b_{i}^{\mathrm{cur}}$ and all boxes in $B_{\mathrm{prev}}^{\left(c_{i}\right)}$, taking the maximum IoU as the temporal consistency score $s_{i}^{\mathrm{IoU}}$:

(11) $$s_{i}^{\mathrm{IoU}}=\max_{b_{k}^{\mathrm{prev}}\in B_{\mathrm{prev}}^{\left(c_{i}\right)}}\mathrm{IoU}\left(b_{i}^{\mathrm{cur}},b_{k}^{\mathrm{prev}}\right)$$

The final confidence score and selection rule are defined as follows:

(12) $$s_{i}^{\mathrm{final}}=s_{i}^{\mathrm{cls}}\times s_{i}^{\mathrm{IoU}}$$

(13) $$M_{j}=\left\{i|s_{i}^{\mathrm{final}}\geq T\right\}$$

where $M_{j}$ represents the set of indices of the current predicted boxes that pass the threshold. Thus, the final pseudo-labels for image $x_{j}^{t}$ are represented as follows:

(14) $${\hat{y}}_{j}^{t}=\left\{\left(b_{i}^{\mathrm{cur}},c_{i}^{\mathrm{cur}}\right)|i\in M_{j}\right\}$$

The proposed strategy effectively identifies predictions with unstable localization, even when these predictions have high classification confidence. By considering predictions across multiple training epochs, our method reduces the noise that may result from a single prediction. Notably, temporal consistency serves as a simple yet effective mechanism to evaluate the quality of bounding box localization without introducing additional computational overhead.

### 3.4. Training Pipeline

Our UDA-OD method follows the training paradigm of AT [4], which involves two stages.

In the warm-up stage, following [4], the student model is trained on labeled source domain data $D_{S}$ using a supervised loss $L_{\sup}$, which combines classification and bounding box regression losses to optimize the model for both tasks. This supervised training establishes a robust initial model. After completing the warm-up, the student model’s parameters are copied to the teacher model, initializing the teacher with identical weights.

In the self-training stage, the teacher model generates high-confidence pseudo-labels for unlabeled target domain data, which guide the student model in learning target-specific features. The teacher model’s parameters are updated through an Exponential Moving Average (EMA) of the student model’s parameters, ensuring stable and gradual refinement.

The total training loss function for the student model in this stage, $L_{\mathrm{total}}$, integrates supervised, unsupervised, and contrastive components to support effective pseudo-label selection, cross-scale prediction consistency, and feature alignment:

(15) $$L_{\mathrm{total}}=\lambda_{\sup}L_{\sup}+\lambda_{\mathrm{unsup}}\left(L_{\mathrm{unsup}}^{I}+L_{\mathrm{unsup}}^{I^{*}}\right)+\lambda_{\mathrm{contra}}L_{\mathrm{contra}}$$

where the factors $\lambda_{\sup}$, $\lambda_{\mathrm{unsup}}$, and $\lambda_{\mathrm{contra}}$ control the influence of each loss component.

This integrated loss function enables the self-training stage to effectively leverage both labeled source and unlabeled target data. Through high-confidence pseudo-labels, cross-scale consistency, and feature alignment, the model achieves robust cross- domain generalization.

## 4. Experiments

### 4.1. Implementation Details

This study employed the Faster-RCNN [12] model with a res-50-FPN backbone and the Detectron2 framework. Experiments were conducted on two NVIDIA RTX 4090 GPUs with a batch size of 4 for both source and target domain data using random sampling. We did not adopt specific data balancing techniques in this study. The network was optimized using SGD with a learning rate of 0.04. We scaled all images by resizing the shorter side of the image to 630 while maintaining the image ratios. Key hyperparameters were set as follows: τ=0.07, $\lambda_{\sup}=1.0$, $\lambda_{\mathrm{unsup}}=1.0$, T=0.6, and $\lambda_{\mathrm{contra}}=0.05$. Other hyperparameters were set to be consistent with [4]. During training, we first performed 10,000 iterations in the warm-up stage, followed by 25,000 iterations in the self-training stage.

We evaluated our method using the standard mean average precision (mAP) metric, with an IoU threshold of 0.5.

Additionally, we used the Source model, trained only on source domain data, as a lower bound, and the Oracle model, trained with full supervision on target domain data, as an upper bound.

### 4.2. Datasets

In this study, we conducted experiments on three typical domain adaptation scenarios for object detection to evaluate the effectiveness of our methods. These scenarios include Cityscapes to Foggy Cityscapes, Sim10k to Cityscapes, and Virtual Mine to Actual Mine. Each scenario presents a unique domain adaptation challenge, covering the adaptation requirements from urban images to foggy environments, synthetic data to real-world data, and from virtual mines to actual mines.

***Cityscapes and Foggy Cityscapes*****:** The Cityscapes dataset [25] consists of high-resolution urban scene images from 50 cities in Germany, primarily intended for semantic segmentation, instance segmentation, and object detection. It contains 5000 finely annotated images, with 2975 for training, 500 for validation, and 1525 for testing. The Foggy Cityscapes dataset [26] was created by applying synthetic fog effects at three density levels (0.02, 0.01, 0.005) to the Cityscapes dataset, simulating visibility under adverse weather conditions. In our experiments, we used the most challenging 0.02 fog level as well as all three fog splits to comprehensively assess model robustness. This domain adaptation task is widely used to evaluate UDA-OD performance under varying weather conditions.

***Sim10k and Cityscapes*****:** The Sim10k dataset [27] contains 10,000 urban scene images generated from a simulated environment, with only the “car” category annotated. This dataset is commonly used for unsupervised domain adaptation from simulation to reality. For domain adaptation, we used the Cityscapes dataset as the target domain, also focusing on the “car” category. This task primarily evaluates the adaptation from synthetic to real data in urban environments.

***Virtual Mine and Actual Mine*****:** The Actual Mine dataset [28] was derived from the public Automine dataset [29], containing 3975 mining-related images, with 2975 for training and 500 for testing. It captures complex lighting conditions, background noise, and diverse target objects, reflecting real mining environments. The Virtual Mine dataset was generated by the PMWorld [30] autonomous simulation platform, simulating virtual scenarios in open-pit mines under various weather and lighting conditions. This platform provides semantic segmentation and bounding box information, resulting in 5000 images with six types of mining-specific objects. Together, these datasets create a virtual-to-real domain adaptation benchmark, effectively validating the model’s adaptability and generalization in complex, real-world mining environments.

### 4.3. Main Results

Extensive experiments across multiple domain adaptation scenarios were conducted and compared with state-of-the-art methods, in order to evaluate the effectiveness of our proposed UDA-OD method. The experimental results, presented in Table 1, Table 2 and Table 3, consistently demonstrate that our method outperforms existing approaches.

#### 4.3.1. Cityscapes to Foggy Cityscapes

We first evaluated our method on the domain adaptation task from Cityscapes to Foggy Cityscapes using two data splits: 0.02 and all data splits. As shown in Table 1, our method achieved the highest mAP, reaching 54.2 and 55.9 on the 0.02 and all splits, respectively.

For the 0.02 split, the source-only model attained an mAP of 30.3, while our method improved this by 24.9 points, reaching 54.2. Furthermore, our method surpassed the best-performing baseline, CMT (mAP 51.1), by 3.1 points and even exceeded the Oracle method (mAP 51.0). In the all split, where the number of unlabeled target domain samples increased, all methods saw performance gains. Nevertheless, our method still surpassed the source-only mAP by 10.3 points, the best previous method, CMT, by 2.6 points, and the Oracle method by 4 points.

In summary, our method consistently enhanced domain adaptation performance across different data splits and weather conditions, validating the effectiveness of the SC and TEPLS strategies. Importantly, our method surpassed the fully supervised Oracle method in both splits, highlighting its potential to significantly reduce annotation costs while maintaining high accuracy.

#### 4.3.2. Sim10k to Cityscapes

For the Sim10k-to-Cityscapes domain adaptation task, our proposed method achieved an AP of 65.8 for the “car” category, surpassing existing methods (Table 2). Compared to the source-only model, the AP was improved by 29.1 points, demonstrating the significant impact of unsupervised domain adaptation on target domain detection performance.

Compared to Deform-DETR-based methods, our method achieved a 3.8-point improvement over the best-performing MRT (62.0 AP), and compared to Faster R-CNN-based methods, it outperformed the best-performing CMT (63.5 AP) by 2.3 points. These results validate the effectiveness of our SC and TEPLS strategies for cross-domain adaptation.

Overall, our method demonstrated substantial improvements in detection accuracy when transferring from synthetic data to real-world scenes, highlighting its potential in complex cross-domain applications.

#### 4.3.3. Virtual Mine to Actual Mine

In the Virtual Mine to Actual Mine domain adaptation task, our method achieved an mAP of 46.8, outperforming all existing methods (as shown in Table 3). The source-only model achieved an mAP of 40.5, while existing methods reached 43.2 mAP with AT and 44.8 mAP with CMT. Through the SC and TEPLS mechanisms, our method improved the mAP to 46.4, and performed particularly well on categories like “Car” (42.5) and “Pushdozer” (77.3), demonstrating robust cross-domain adaptation.

Overall, our method showed strong generalization in this transfer task, significantly improving detection accuracy from synthetic data to real mine environments by optimizing pseudo-labels and enhancing multi-scale feature consistency. This approach effectively addresses unstructured, complex scenarios, highlighting its potential for real-world applications.

### 4.4. Component and Parameter Ablation Analysis

In this section, we conducted a comprehensive ablation analysis on both the model components and the parameters.

#### 4.4.1. Component Ablation Analysis

To assess the impact of each component, we conducted ablation studies on CSPC, ICFC, and the TEPLS strategy for the domain adaptation task from Cityscapes to Foggy Cityscapes. The results are shown in Table 4.

The baseline model had an mAP of 50.2. Adding $L_{\mathrm{RPN}}^{I^{*}}$ to the CSPC boosted the mAP to 52.1, indicating the importance of using pseudo-labels to guide RPN training at both scales. Further adding a second $L_{\mathrm{RPN}}^{I^{*}}$ to the CSPC improved the mAP to 53.5, showing that including pseudo-labels to guide ROI training at both scales can enhance performance. Introducing $L_{\mathrm{contra}}$ from ICFC raised the mAP to 53.8, demonstrating enhanced feature consistency between scales. Finally, adding TEPLS raised the mAP to 54.2, showing its importance for enhancing pseudo-labeling stability and training effectiveness. These results illustrate the benefits of combining temporal consistency with multi-scale learning for unsupervised domain adaptation in object detection.

#### 4.4.2. Parameter Ablation Analysis

We also performed parameter ablation studies to evaluate the effect of different pseudo-label thresholds on overall mAP. The results for various thresholds are shown in Table 5.

The results show that pseudo-label thresholding significantly affects model performance. At a threshold of 0.5, the model’s mAP was 53.2, indicating that the inclusion of noisy pseudo-labels hindered performance. Increasing the threshold to 0.6 achieved the highest mAP of 54.2, balancing high-quality pseudo-labels with noise reduction. Further increasing the threshold to 0.7 and 0.8 caused a slight decline in mAP, as fewer pseudo-labels reduced the training signal.

### 4.5. Comprehensive Analysis

This section provide a comprehensive analysis of our proposed method, combining qualitative and visual assessments to deepen understanding of the model’s domain adaptation capabilities. We evaluated and compared pseudo-label selection strategies, track performance changes for small objects across iterations, visualize feature alignment with t-SNE, and assess detection quality visually. These analyses collectively demonstrate our approach’s effectiveness in enhancing cross-domain object detection.

#### 4.5.1. Qualitative Comparison of Pseudo-Label Selection

**Figure 2:**
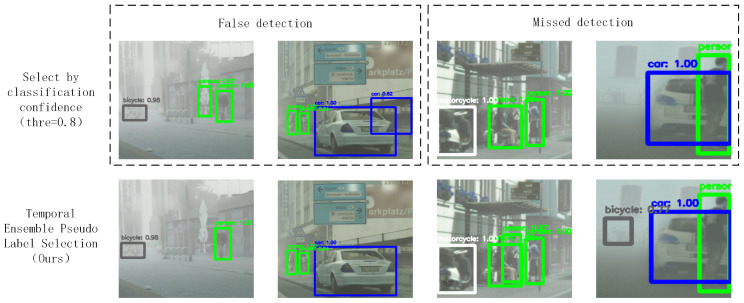
Qualitative comparison of pseudo-label selection strategies. The top row shows results using only classification confidence (threshold = 0.8), leading to false detections (**left**) and missed detections (**right**). The bottom row shows results using our proposed TEPLS strategy.

To evaluate the performance of the common classification confidence-based pseudo-label selection approach and the TEPLS strategy proposed in this paper, we visually compared their pseudo-labels in complex detection tasks (Figure 2).

The results show that the confidence-based strategy tended to rely on high-confidence predictions within a single frame, leading to false positives and missed detections. For example, it falsely detected “people” and “car” and missed “bicycle”. By incorporating temporal ensemble information, TEPLS significantly reduced such errors, improving the accuracy and stability of pseudo-labels by combining multi-frame detection results. This approach effectively filters single-frame noise and reduces both false positives and missed detections.

#### 4.5.2. AP Comparison over Iterations

**Figure 3:**
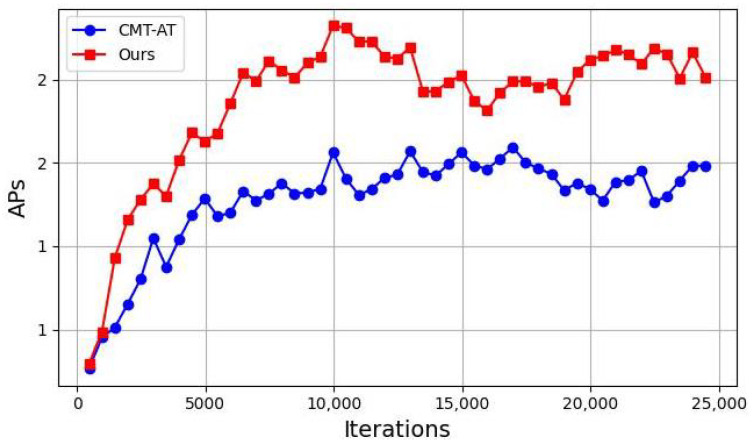
Comparison of average precision for small objects (AP) over iterations.

To evaluate the effect of our proposed method on small object detection performance, we tracked the average precision for small objects (AP) over training iterations. Figure 3 compares our method with the baseline method CMT-AT.

As shown in Figure 3, our method consistently outperforms CMT-AT in small object detection across all iterations, with particularly strong improvement in the early stages. This indicates that our approach accelerates the learning of small object representations.

#### 4.5.3. Feature Visualization

**Figure 4:**
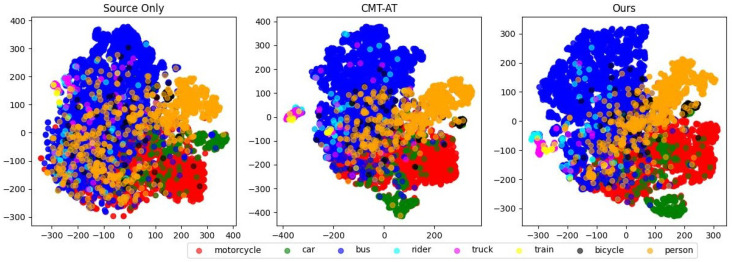
t-SNE visualization of feature distributions for different approaches: Source Only (**left**), CMT-AT (**middle**), and our proposed method (**right**). Each point represents an object feature, with different colors for different object classes.

To further examine our method’s impact, we used t-SNE to visualize the feature distributions of different domain adaptation methods. Figure 4 shows t-SNE visualizations for three settings: source-only training, CMT-AT, and our proposed method.

Figure 4 shows that our method forms tighter and more distinct clusters for each category, indicating improved feature alignment and class separability. The source-only training exhibits considerable overlap between categories, indicating limited domain adaptation. CMT-AT shows moderate improvement, while our approach achieves clearer boundaries between categories, enhancing feature discriminability and target domain detection performance.

#### 4.5.4. Model Efficiency Evaluation

Table 6 compares the core efficiency metrics of several models, providing a comprehensive assessment of the performance–resource trade-off for each model.

For inference time, like the AT and CMT approaches, our approach resulted in a model with the same inference speed as the original object detection model (e.g., Faster R-CNN), with an inference time of 36 ms in both cases. This is because we adopted the TEPL strategy to compute localization quality via boxes at different training epochs without modifying the network structure of the teacher model.

In terms of training resource consumption, our method had small increases in training time (3.75 h) and memory consumption (6582 MB) compared to AT (3.35 h, 5983 MB) and CMT (3.50 h, 6094 MB). The increased training time is acceptable compared to the significant improvement in model performance since UDA-OD training is typically performed in cloud or offline environments with strong computational power.

In summary, despite the increases in training time and GPU memory requirements, our UDA-OD method provides substantial performance gains over Faster R-CNN and demonstrates a good balance of efficiency and performance compared to other UDA-OD methods.

#### 4.5.5. Qualitative Evaluation

**Figure 5:**
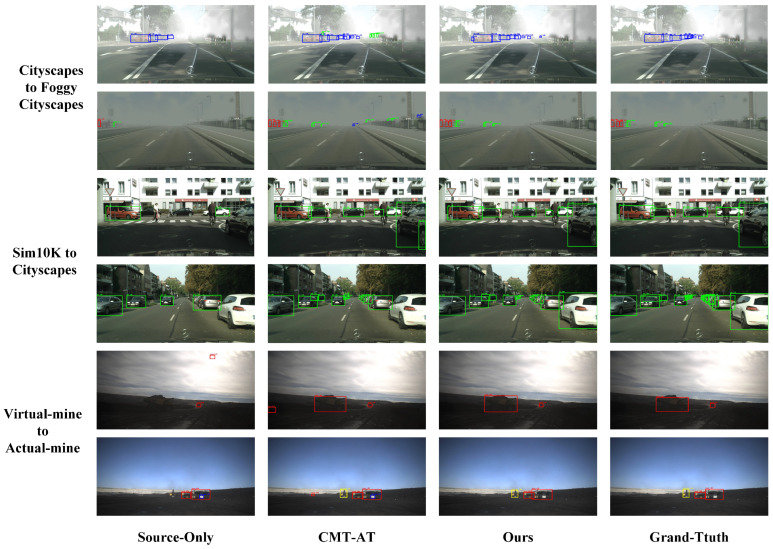
Qualitative results of domain adaptation on different benchmark datasets. The figure shows adaptation scenarios from Cityscapes to Foggy Cityscapes, Sim10K to Cityscapes, and Virtual Mine to Actual Mine. Columns represent different approaches: Source-Only, CMT-AT, Ours, and the corresponding Ground-Truth. Our proposed method consistently produces detection results closer to the Ground-Truth compared to both Source-Only and CMT-AT, demonstrating improved adaptation performance across diverse target domains.

To assess practical performance in unsupervised domain adaptation, Figure 5 shows the cross-domain detection results of different methods, including Source-Only, CMT-AT, our method, and ground-truth annotations across three cross-domain tasks. This comparison highlights each method’s effectiveness in handling domain shifts and environmental complexities.

The results indicate that the Source-Only model performed poorly in cross-domain tasks, frequently exhibiting false positives and missed detections. CMT-AT showed some improvements but remains unstable in challenging conditions such as adverse weather and complex lighting. In contrast, our method consistently achieved results closer to the ground truth, demonstrating significant advantages across all tasks. Specifically, in foggy weather, complex traffic scenes, and low-contrast mining environments, our method accurately detected target objects, showcasing enhanced robustness and adaptability.

## 5. Discussion

While our proposed method enhances unsupervised domain adaptation for object detection, it still has some limitations. First, although the TEPLS strategy improves pseudo-label accuracy, it applies a uniform threshold across all categories. This uniformity may not account for the varying detection difficulties and confidence distributions among different object classes, potentially leading to suboptimal pseudo-label selection for some categories. For instance, in the Virtual Mine to Actual Mine domain adaptation task, the AP for the “wide body” and “water car” categories was significantly lower compared to other classes, highlighting the limitations of a fixed threshold.

Second, in enforcing scale consistency, we utilized a fixed downsampling ratio to create multi-scale inputs. This fixed ratio limited the diversity of scale variations during training, which may have hindered the model’s ability to generalize to objects at scales not represented by the chosen downsampling factor.

To address these limitations, future work will explore adaptive thresholding strategies for pseudo-label selection, allowing thresholds to be adjusted based on class-specific characteristics or confidence distributions. Additionally, we plan to incorporate variable downsampling ratios or multi-scale training techniques to enhance scale diversity, thereby improving the model’s robustness to a wider range of object sizes.

Building on these improvements, we also aim to enhance the practicality of our proposed UDA-OD method for real-world applications such as autonomous driving and industrial automation to improve its performance in scenarios with significant domain shifts. For instance, data transfer is especially critical in scenarios like transitioning from normal lighting to dark or dusty environments [43], or transferring simulated images to real ones [28]. These scenarios often face challenges such as limited target domain data volume or diversity, difficulty in collection, and high labeling costs. Currently, the performance of our method in these scenarios still lags behind fully supervised target domain training models. Therefore, we plan to incorporate active learning to select the most representative target domain samples for labeling, and then investigate the semi-supervised object detection domain adaptation problem under a small number of target domain labels. This approach aims to achieve high-performance object detection in the target domain with low labeling cost, thereby enhancing the practicality and operability of the method.

## 6. Conclusions

This paper presents an effective UDA-OD that addresses key challenges in cross-domain scenarios. Our approach combines Cross-Scale Prediction Consistency (CSPC), Intra-Class Feature Consistency (ICFC), and Temporal Ensemble Pseudo-Label Selection (TEPLS) to enhance detection performance across scales and improve pseudo-label stability. CSPC ensures robust detection of small objects through multi-resolution supervision, ICFC aligns object features within each class using contrastive learning, and TEPLS refines pseudo-label quality by integrating temporal consistency with classification confidence.

Experimental results on benchmarks such as Cityscapes to Foggy Cityscapes, Sim10k to Cityscapes, and Virtual Mine to Actual Mine demonstrate that our method outperforms existing UDA-OD approaches, achieving higher accuracy and improved adaptation stability. The proposed framework effectively leverages unlabeled target data, enhancing both detection robustness and model generalization across domains.

## Figures and Tables

**Table 1:** Performance comparison of different methods in unsupervised domain adaptation from Cityscapes to Foggy Cityscapes. The table shows mean average precision (mAP) and per-category AP scores for various models across two training dataset splits: “0.02” split and “all” split. In the “Backbone” column, R50-FPN refers to ResNet-50 with Feature Pyramid Network [31], R50 is ResNet-50, and V16 denotes VGG-16. The “Detector” column includes Deform-DETR (Deformable Detection Transformer [18]) and Faster R-CNN.

Method	Backbone	Detector	Split	Bicycle	Bus	Car	Motorcycle	Person	Rider	Train	Truck	mAP
Source	R50-FPN	Faster R-CNN	0.02	38.4	27.5	44.9	29.3	40.4	41.7	1.9	18.1	30.3
Oracle	R50-FPN	Faster R-CNN	0.02	51.2	52.7	74.1	42.0	56.7	57.3	39.7	35.4	51.0
AT [4]	V16	Faster R-CNN	0.02	51.3	64.9	63.6	42.1	45.3	55.7	34.9	36.8	49.3
CMT [5]	V16	Faster R-CNN	0.02	51.2	66.0	63.7	41.4	45.9	55.7	38.8	39.6	50.3
MOTOR [32]	R50	Faster R-CNN	0.02	35.6	38.6	44.0	28.3	30.6	41.4	40.6	21.9	35.1
SFA [33]	R50	Deform-Detr	0.02	44.0	46.2	62.6	28.3	46.5	48.6	29.4	25.1	41.3
MTTrans [34]	R50	Deform-Detr	0.02	46.5	45.9	65.2	32.6	47.7	49.9	33.8	25.8	43.4
DA-DETR [35]	R50	Deform-Detr	0.02	46.5	45.9	63.1	32.6	49.9	50.0	33.8	25.8	43.5
$O^{2}$Net [36]	R50	Deform-Detr	0.02	45.9	47.6	63.6	38.0	48.7	51.5	47.8	31.1	46.8
AQT [37]	R50	Deform-Detr	0.02	46.4	53.7	64.4	36.0	49.3	52.3	44.7	27.7	47.1
MTM [38]	R50	Deform-Detr	0.02	47.7	54.4	67.2	38.4	51.0	53.4	41.6	37.2	48.9
TDD [39]	R50	Faster R-CNN	0.02	49.1	51.1	64.1	38.9	50.7	53.7	37.5	36.3	49.2
MRT [40]	R50	Faster R-CNN	0.02	47.1	58.1	68.7	41.0	52.8	51.7	54.5	35.9	51.2
SA-DA-Faster [7]	R50-FPN	Faster R-CNN	0.02	45.4	50.3	62.1	32.4	48.5	52.6	31.5	29.5	44.0
AT [4]	R50-FPN	Faster R-CNN	0.02	53.3	52.1	66.0	41.1	51.2	57.8	45.4	34.7	50.2
CMT [5]	R50-FPN	Faster R-CNN	0.02	53.1	55.0	66.7	39.2	51.0	57.2	51.9	34.7	51.1
Ours	R50-FPN	Faster R-CNN	0.02	57.7	57.5	69.0	43.6	52.9	60.5	52.2	40.4	54.2
Source	R50-FPN	Faster R-CNN	All	50.8	46.7	62.4	37.0	50.6	54.8	31.5	31.1	45.6
Oracle	R50-FPN	Faster R-CNN	All	55.0	57.9	72.2	39.1	53.5	58.8	43.6	34.9	51.9
PDA [41]	V16	Faster R-CNN	All	35.9	44.1	54.4	29.1	36.0	45.5	25.8	24.3	36.9
ICR-CCR [42]	V16	Faster R-CNN	All	34.6	36.4	49.2	30.3	32.9	43.8	36.4	27.2	37.4
AT [4]	R50-FPN	Faster R-CNN	All	53.1	59.0	69.5	43.1	55.2	59.3	46.2	40.7	53.2
CMT [5]	R50-FPN	Faster R-CNN	All	53.6	58.6	69.9	42.5	54.5	58.9	47.3	41.3	53.3
Ours	R50-FPN	Faster R-CNN	All	53.0	63.0	71.9	44.4	56.4	61.2	55.4	42.2	55.9

**Table 2:** Performance comparison of different methods in domain adaptation from Sim10k to Cityscapes.

Method	Backbone	Detector	AP (Car)
Source	R50-FPN	Faster R-CNN	36.7
Oracle	R50-FPN	Faster R-CNN	78.0
AT [4]	V16	Faster R-CNN	55.1
TDD [39]	R50	Faster R-CNN	63.3
MOTOR [32]	R50	Faster R-CNN	46.6
SFA [33]	R50	Deform-Detr	52.6
AQT [37]	R50	Deform-Detr	53.4
$O^{2}$Net [36]	R50	Deform-Detr	54.1
MTTrans [34]	R50	Deform-Detr	57.9
MRT [40]	R50	Deform-Detr	62.0
SA-DA-Faster [7]	R50-FPN	Faster R-CNN	55.8
AT [4]	R50-FPN	Faster R-CNN	62.0
CMT [5]	R50-FPN	Faster R-CNN	63.5
Ours	R50-FPN	Faster R-CNN	65.8

**Table 3:** Performance comparison of different methods in domain adaptation from Virtual Mine to Actual Mine.

Method	Backbone	Detector	Pushdozer	Car	Excavator	Truck	Water_car	Widebody	mAP
Source	R50-FPN	Faster R-CNN	44.3	29.9	41.3	71.0	4.3	4.4	40.5
Oracle	R50-FPN	Faster R-CNN	98.3	90.4	87.5	95.2	85.1	87.5	90.7
AT [4]	R50-FPN	Faster R-CNN	75.0	22.7	78.0	80.6	1.2	1.5	43.2
CMT [5]	R50-FPN	Faster R-CNN	76.9	40.0	70.7	76.2	0.5	1.6	44.3
Ours	R50-FPN	Faster R-CNN	77.3	42.5	77.7	79.6	1.4	2.3	46.8

**Table 4:** Ablation studies on key components.

Baseline	CSPC		ICFC	TEPLS	mAP
$L_{\sup}+L_{\mathrm{unsup}}^{I}$	** $L_{\mathrm{RPN}}^{I^{*}}$ **	** $L_{\mathrm{RPN}}^{I^{*}}$ **	** $L_{\mathrm{contra}}$ **	-	
✓					50.2
✓	✓				52.1
✓	✓	✓			53.5
✓	✓	✓	✓		53.8
✓	✓	✓	✓	✓	54.2

**Table 5:** Parameter ablation studies on the effect of different pseudo-label threshold values.

Threshold	0.5	0.6	0.7	0.8
mAP	53.2	54.2	54.0	53.7

**Table 6:** Comparison of model efficiency metrics.

Model	Training Duration (h)	Inference Time (ms)	GPU Memory Consumption (MB)
Faster R-CNN [12]	2.00	36	3667
AT [4]	3.35	36	5983
CMT [5]	3.50	36	6094
Ours Method	3.75	36	6582

## Data Availability

Data are available in a publicly accessible repository that does not issue DOIs. Cityscapes, Foggy Cityscapes, and Sim10k are publicly available datasets and were analyzed in this study. These datasets can be accessed here: https://www.cityscapes-dataset.com (Cityscapes, Foggy Cityscapes) and https://fcav.engin.umich.edu/projects/driving-in-the-matrix (Sim10k). Both were accessed on 1 May 2024. The Virtual Mine and Actual Mine datasets were constructed in our previous work. These datasets are private and can be available upon request from the corresponding author.

## References

[1] Zou Z., Chen K., Shi Z., Guo Y., Ye J. (2023). Object detection in 20 years: A survey. Proc. IEEE.

[2] Chen Y., Li W., Sakaridis C., Dai D., Van Gool L. Domain Adaptive Faster R-CNN for Object Detection in the Wild. Proceedings of the IEEE Conference on Computer Vision and Pattern Recognition (CVPR).

[3] Oza P., Sindagi V.A., Sharmini V.V., Patel V.M. (2023). Unsupervised domain adaptation of object detectors: A survey. IEEE Trans. Pattern Anal. Mach. Intell..

[4] Li Y.-J., Dai X., Ma C.-Y., Liu Y.-C., Chen K., Wu B., He Z., Kitani K., Vajda P. Cross-domain Adaptive Teacher for Object Detection. Proceedings of the IEEE Conference on Computer Vision and Pattern Recognition (CVPR).

[5] Cao S., Joshi D., Gui L.-Y., Wang Y.-X. Contrastive Mean Teacher for Domain Adaptive Object Detectors. Proceedings of the IEEE Conference on Computer Vision and Pattern Recognition (CVPR).

[6] Liu X., Zhang B., Liu N. (2023). Cross-domain object detection by dual adaptive branch. Sensors.

[7] Chen Y., Wang H., Li W., Sakaridis C., Dai D., Van Gool L. (2021). Scale-aware domain adaptive faster R-CNN. Int. J. Comput. Vis..

[8] Zhang L., Zhou W., Fan H., Luo T., Ling H. (2024). Robust Domain Adaptive Object Detection with Unified Multi-Granularity Alignment. IEEE Trans. Pattern Anal. Mach. Intell..

[9] Xu M., Zhang Z., Hu H., Wang J., Wang L., Wei F., Bai X., Liu Z. End-to-End Semi-Supervised Object Detection with Soft Teacher. Proceedings of the IEEE/CVF International Conference on Computer Vision (ICCV).

[10] He Y., Chen W., Liang K., Tan Y., Liang Z., Guo Y. (2023). Pseudo-label correction and learning for semi-supervised object detection. arXiv.

[11] Mattolin G., Zanella L., Ricci E., Wang Y. ConfMix: Unsupervised Domain Adaptation for Object Detection via Confidence-based Mixing. Proceedings of the IEEE/CVF Winter Conference on Applications of Computer Vision (WACV).

[12] Ren S., He K., Girshick R., Sun J. (2016). Faster R-CNN: Towards Real-Time Object Detection with Region Proposal Networks. IEEE Trans. Pattern Anal. Mach. Intell..

[13] Liu W., Anguelov D., Erhan D., Szegedy C., Reed S., Fu C.-Y., Berg A.C. SSD: Single Shot Multibox Detector. Proceedings of the Computer Vision—ECCV 2016: 14th European Conference.

[14] Redmon J., Farhadi A. (2018). YOLOv3: An incremental improvement. Proceedings of the IEEE Conference on Computer Vision and Pattern Recognition (CVPR).

[15] Law H., Deng J. CornerNet: Detecting Objects as Paired Keypoints. Proceedings of the European Conference on Computer Vision (ECCV).

[16] Tian Z., Shen C., Chen H., He T. (2020). FCOS: A simple and strong anchor-free object detector. IEEE Trans. Pattern Anal. Mach. Intell..

[17] Carion N., Massa F., Synnaeve G., Usunier N., Kirillov A., Zagoruyko S. End-to-end object detection with transformers. Proceedings of the European Conference on Computer Vision (ECCV).

[18] Zhu X., Su W., Lu L., Li B., Wang X., Dai J. Deformable DETR: Deformable transformers for end-to-end object detection. Proceedings of the ICLR 2021.

[19] Zhang H., Li F., Liu S., Zhang L., Su H., Zhu J., Ni L.M., Shum H.-Y. (2022). DINO: DETR with Improved Denoising Anchor Boxes for End-to-End Object Detection. arXiv.

[20] Sun P., Zhang R., Jiang Y., Kong T., Xu C., Zhan W., Tomizuka M., Li L., Yuan Z., Wang C. Sparse R-CNN: End-to-End Object Detection with Learnable Proposals. Proceedings of the IEEE/CVF Conference on Computer Vision and Pattern Recognition (CVPR).

[21] Liang T., Bao H., Pan W., Pan F. (2022). Traffic Sign Detection via Improved Sparse R-CNN for Autonomous Vehicles. J. Adv. Transp..

[22] Arruda V.F., Berriel R.F., Paixão T.M., Badue C., De Souza A.F., Sebe N., Oliveira-Santos T. (2022). Cross-Domain Object Detection Using Unsupervised Image Translation. Expert Syst. Appl..

[23] Zhang G., Wang L., Chen Z. (2024). A Step-Wise Domain Adaptation Detection Transformer for Object Detection under Poor Visibility Conditions. Remote Sens..

[24] Zhuang C., Han X., Huang W., Scott M. IFAN: Image-Instance Full Alignment Networks for Adaptive Object Detection. Proceedings of the AAAI Conference on Artificial Intelligence.

[25] Cordts M., Omran M., Ramos S., Rehfeld T., Enzweiler M., Benenson R., Franke U., Roth S., Schiele B. The Cityscapes Dataset for Semantic Urban Scene Understanding. Proceedings of the IEEE Conference on Computer Vision and Pattern Recognition (CVPR).

[26] Sakaridis C., Dai D., Van Gool L. (2018). Semantic foggy scene understanding with synthetic data. Int. J. Comput. Vis..

[27] Johnson-Roberson M., Barto C., Mehta R., Sridhar S.N., Rosaen K., Vasudevan R. (2016). Driving in the Matrix: Can Virtual Worlds Replace Human-Generated Annotations for Real World Tasks?. arXiv.

[28] Guo L., Guo Y., Ai Y., Ge S. (2024). Active Parallel Teacher for Human-in-the-Loop Sim2Real Object Detection in Autonomous Haulage Trucks. IEEE Trans. Intell. Veh..

[29] Li Y., Li Z., Teng S., Zhang Y., Zhou Y., Zhu Y. AutoMine: An Unmanned Mine Dataset. Proceedings of the IEEE Conference on Computer Vision and Pattern Recognition (CVPR).

[30] Ai Y., Liu Y., Gao Y., Zhao C., Cheng X., Han J., Tian B., Chen L., Wang F.-Y. (2024). PMWorld: A Parallel Testing Platform for Autonomous Driving in Mines. IEEE Trans. Intell. Veh..

[31] Lin T.Y., Dollár P., Girshick R., He K., Hariharan B., Belongie S. Feature Pyramid Networks for Object Detection. Proceedings of the IEEE Conference on Computer Vision and Pattern Recognition (CVPR).

[32] Cai Q., Pan Y., Ngo C.W., Tian X., Duan L., Yao T. Exploring Object Relation in Mean Teacher for Cross-Domain Detection. Proceedings of the IEEE/CVF Conference on Computer Vision and Pattern Recognition (CVPR).

[33] Wang W., Cao Y., Zhang J., He F., Zha Z.J., Wen Y., Tao D. Exploring Sequence Feature Alignment for Domain Adaptive Detection Transformers. Proceedings of the 29th ACM International Conference on Multimedia.

[34] Yu J., Liu J., Wei X., Zhou H., Nakata Y., Gudovskiy D., Okuno T., Li J., Keutzer K., Zhang S. (2022). MTTrans: Cross-domain Object Detection with Mean Teacher Transformer. Proceedings of the Computer Vision—ECCV 2022: 17th European Conference.

[35] Zhang J., Huang J., Luo Z., Zhang G., Zhang X., Lu S. DA-DETR: Domain Adaptive Detection Transformer With Information Fusion. Proceedings of the IEEE/CVF Conference on Computer Vision and Pattern Recognition (CVPR).

[36] Gong K., Li S., Li S., Zhang R., Liu C.H., Chen Q. Improving Transferability for Domain Adaptive Detection Transformers. Proceedings of the 30th ACM International Conference on Multimedia.

[37] Huang W.J., Lu Y.L., Lin S.Y., Xie Y., Lin Y.Y. AQT: Adversarial Query Transformers for Domain Adaptive Object Detection. Proceedings of the 31st International Joint Conference on Artificial Intelligence (IJCAI).

[38] Weng W., Yuan C. Mean Teacher DETR with Masked Feature Alignment: A Robust Domain Adaptive Detection Transformer Framework. Proceedings of the AAAI Conference on Artificial Intelligence.

[39] He M., Wang Y., Wu J., Wang Y., Li H., Li B., Gan W., Wu W., Qiao Y. Cross Domain Object Detection by Target-Perceived Dual Branch Distillation. Proceedings of the IEEE/CVF Conference on Computer Vision and Pattern Recognition (CVPR).

[40] Zhao Z., Wei S., Chen Q., Li D., Yang Y., Peng Y. Masked retraining teacher-student framework for domain adaptive object detection. Proceedings of the IEEE/CVF International Conference on Computer Vision.

[41] Hsu H.K., Yao C.H., Tsai Y.H., Hung W.C., Tseng H.Y., Singh M., Yang M.H. Progressive Domain Adaptation for Object Detection. Proceedings of the IEEE Winter Conference on Applications of Computer Vision (WACV).

[42] Wu A., Li W., Zhou W., Cao X. Vector-Decomposed Disentanglement for Domain-Invariant Object Detection. Proceedings of the IEEE/CVF International Conference on Computer Vision (ICCV).

[43] Ma S., Pan W., Li N., Du S., Liu H., Xu B., Xu C., Li X. (2024). Low-Light Image Enhancement Using Retinex-Based Network with Attention Mechanism. Int. J. Adv. Comput. Sci. Appl..

